# All-Solid-State Optical Phased Arrays of Mid-Infrared Based Graphene-Metal Hybrid Metasurfaces

**DOI:** 10.3390/nano11061552

**Published:** 2021-06-11

**Authors:** Yue Wang, Yu Wang, Guohui Yang, Qingyan Li, Yu Zhang, Shiyu Yan, Chunhui Wang

**Affiliations:** 1 National Key Laboratory of Tunable Laser Technology, Harbin Institute of Technology, Harbin 150001, China; seraph@hit.edu.cn (Y.W.); yvonne@hit.edu.cn (Y.W.); tom_li@cc.acmlife.org (Q.L.); 18B921023@stu.hit.edu.cn (Y.Z.); meloyan@hit.edu.cn (S.Y.); 2 Shenzhen Glint Institute of AI and Robotics, Shenzhen 518057, China; 3 School of Electronic and Information Engineering, Harbin Institute of Technology, Harbin 150001, China; gh.yang@hit.edu.cn

**Keywords:** optical phased arrays, graphene, metasurface, beam steering

## Abstract

Optical phased arrays (OPAs) are essential optical elements in applications that require the ability to manipulate the light-wavefront, such as beam focusing and light steering. To miniaturize the optical components, active metasurfaces, especially graphene metasurfaces, are used as competent alternatives. However, the metasurface cannot achieve strong resonance effect and phase control function in the mid-infrared region only relying on a single-layer graphene. Here we present a graphene-metal hybrid metasurface that can generate a specific phase or a continuous sweep in the range of a 275°-based single-layer graphene structure. A key feature of our design is that the phase adjustment mainly depends on the combination mechanism of resonance intensity and frequency modulation. An all-solid-state, electrically tunable, and reflective OPA is designed by applying the bias voltage to a different pixel metasurface. The simulation results show that the maximum deflection angle of the OPA can reach 42.716°, and the angular resolution can reach 0.62°. This design can be widely applied to mid-infrared imaging, optical sensing, and optical communication systems.

## 1. Introduction

OPAs [1,2,3,4,5,6,7,8,9] are utilized to focus and steer light and serve as the components of many applications, such as light detection and ranging (LiDAR) [10,11], metalens [12,13], and optical communication systems [14]. OPAs can control beam deflection without mechanical parts, which can make the emitted light or reflected light enhanced in some directions and reduced in other directions by changing the phase of the unit structure. The OPAs in the microwave [15,16] and near-infrared [17,18,19] spectra have been studied extensively by researchers. However, there are few reports of OPAs in the mid-infrared(MIR) band, which can be widely used in chemical and biological sensing, free-space communication, and infrared countermeasures [20].

A metasurface [21,22,23,24,25,26,27,28,29] regulates the amplitude, phase, and polarization of incident electromagnetic or light waves by designing the shape and size of the medium surface and serving as photonic integration and high-performance optical devices. For the vast majority of metasurfaces, the characteristics are defined and fixed at the time of design and fabrication. In order to solve this problem, researchers have proposed tunable metasurfaces by adding variable materials, semiconductor materials, ferroelectric materials, and other tunable materials into traditional metasurfaces. The characteristics of a metasurface can be controlled by applied voltage [30,31,32,33], optical pumping [34,35], changing temperature [36], and changing mechanical structure [37,38]. Among these tuning mechanisms, applied voltage modulation is one of the preferred solutions for active metasurfaces due to the advantages of high speed, flexibility, and reversibility. A recent study proposed a use of voltage modulation in which the ITO material was embedded in the metasurface. With this approach, all-solid-state OPAs for three-dimensional LiDAR applications were reported [39].

Graphene, which is a new type of two-dimensional material, has emerged as a tuning material for active metamaterial and active metasurfaces through carrier concentration and surface conductivity, is controlled by applied voltage [40,41,42,43]. Compared with ITO [12,44] and liquid crystal [45] materials, it has strong stability and high modulation speed. Multilayer graphene-dielectric metasurfaces have been demonstrated to achieve tunable MIR beam steering [46,47]. The wide range of output angles mainly depends on the effective refractive index, and is manipulated by changing the chemical potential of each graphene layer. Most of the OPA devices based on metasurfaces are realized by stacking multilayer graphene [46,48,49]. M. Beruete et al. [46] demonstrated tunable MIR beam steering devices based on multilayer graphene-dielectric metamaterials. In order to solve the complex problem of preparing multilayer graphene, Jianbo Yin et al. [50] design a tunable terahertz metasurface which consists of two different trapezoid graphene ribbons. By changing the Fermi level of the graphene ribbons, this metasurface can cover a nearly 2π phase shift and control terahertz waves. However, the metasurface cannot achieve a strong resonance effect and phase control function only relying on the single-layer graphene, while the manufacturing problem of multilayer graphene cannot be well solved in the MIR band. Here, we present an all-solid-state OPA composed of a graphene-metal hybrid metasurface designed to demonstrate continuous beam deflection by controlling the applied bias voltage in the MIR band. Detailed numerical investigations revealed that the phase adjustment range of the metasurface could reach 275°. The OPA, which is combined with a metasurface array, can achieve maximum beam deflection with an angle of 42.716° in the MIR by utilizing the voltage of each column unit. In addition, the resolution of the OPA can be increased to 0.62° by increasing the metasurface size. The proposed all-solid-state OPA can be used in tunable transmitter modules or receiver modules for MIR imaging and sensing.

## 2. Structure and Methods

**Figure 1:**
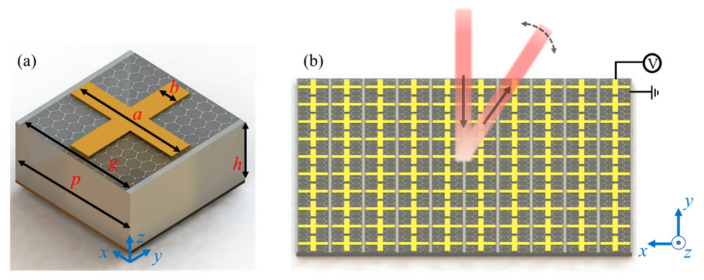
(**a**) Sketch of the unit cell for the proposed active graphene-metal hybrid metasurface. The structural parameters include *a* = 2.1, *b* = 0.4, *g* = 2.3, *p* = 2.5, and *h* = 0.23 μm. (**b**) OPA configuration-based metasurface array.

Figure 1a shows the proposed metasurface unit cell structure diagram. From top to bottom, the demonstrated structural system consists of a graphene-metal hybrid pattern, a silicon dioxide (SiO_2_) layer, and a copper (Cu) layer. The metal pattern was selected as the same Cu material as the substrate and designed as a cross shape to facilitate future batch preparation and engineering implementation. The period of this structure in the *x* and *y* directions was *p* = 2.5 μm. The geometric parameters of the top layer were *a* = 2.1, *b* = 0.4 and *g* = 2.3 μm. The thickness of the SiO_2_ layer, the cross metal, and the copper substrate were *h* = 0.23, *t*_1_ = 0.01, and *t*_2_ = 0.01 μm. Figure 1b shows the OPA architecture, in which the metal substrate is applied to the graphene strip and the bias voltage is applied to the graphene strip. The cross structure at the tail of each column can be used to replace the graphene strip to simplify the preparation and stabilize operation.

The calculations were carried out using the frequency domain solver in electromagnetic full-wave tools CST Microwave Studio. Moreover, the plane wave normally impinges on the metasurface, composed of periodic elements shown in Figure 1a, along the *z*-direction with the *E_x_*-polarization. The graphene was characterized by complex surface conductivity calculated using the Kubo formula without magnetic field bias [51]:

(1) $$\sigma (\omega ,\mu_{c})=\frac{ie^{2}(\omega +i2\Gamma )}{\pi \hbar^{2}}\cdot \left[\begin{array}{c} \frac{1}{{\left(\omega +i2\Gamma \right)}^{2}}\int_{0}^{\infty }\varepsilon \left(\frac{\partial f_{d}(\varepsilon )}{\partial \varepsilon }\right)d\varepsilon \\ -\int_{0}^{\infty }\varepsilon \left(\frac{f_{d}(-\varepsilon )-f_{d}(\varepsilon )}{{\left(\omega +i2\Gamma \right)}^{2}-4\left(\varepsilon /\hbar^{2}\right)}\right)d\varepsilon \end{array}\right]$$

(2) $$f_{d}(\varepsilon )={\left[\exp\left(\varepsilon -\mu_{c}\right)/\left(k_{B}T\right)+1\right]}^{-1},$$

where *e* is the charge of an electron, *ε* is the energy of the incident wave, *ω* is the angular frequency, *k*_B_ is Boltzmann’s constant, *ħ* is the reduced Planck’s constant, *f*_d_(*ε*) is the Fermi–Dirac distribution, *Γ* is the phenomenological scattering rate, *μ*_c_ is the chemical potential, and *T* is the temperature.

In addition, the relationship between the Fermi level of graphene and the applied bias voltage can be expressed as

(3) $$\frac{\varepsilon_{0}\pi \hbar^{2}\nu_{F}^{2}}{e}E_{\mathrm{bias}}=\int_{0}^{\infty }\varepsilon \left[f_{d}\left(\varepsilon \right)-f_{d}\left(\varepsilon +2\mu_{c}\right)\right]d\varepsilon ,$$

where *E*_bias_ is the bias voltage and *ν*_F_ is the electron speed. To simulate the properties of the graphene material, it was regarded as a thin layer of 0.001 µm thickness and its complex surface conductivity was calculated according to Equations (1) and (2).

## 3. Results and Analyses

**Figure 2:**
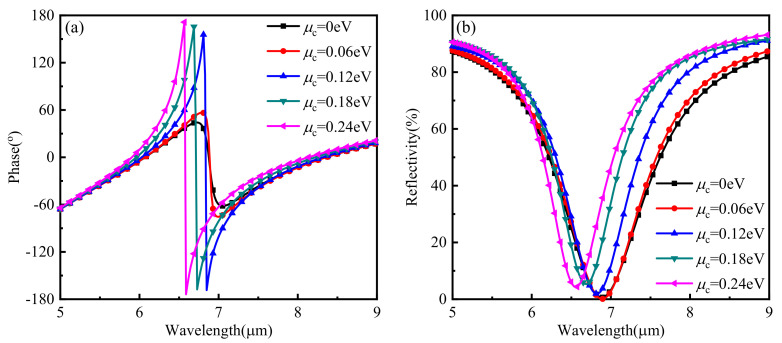
(**a**) Reflection phase performances with various Fermi levels of graphene. (**b**) Reflection amplitude performances with various Fermi levels of graphene.

Firstly, the phase and reflection curves of the hybrid structure were calculated. Figure 2 shows that the abrupt phase change occurred at 6.9 μm. The strong resonance effect also emerged at this wavelength when the Fermi level of graphene was 0 eV. The center wavelength would be shifted to 6.8 μm, which was accompanied by a more obvious abrupt phase change and a more substantial resonance effect when the Fermi level of the graphene increased to 0.12 eV. Furthermore, subsequent simulation results showed that the center wavelength would continue to blue shift while the resonance effect would also be changed accordingly at the same time if the Fermi level of graphene continued to increase.

**Figure 3:**
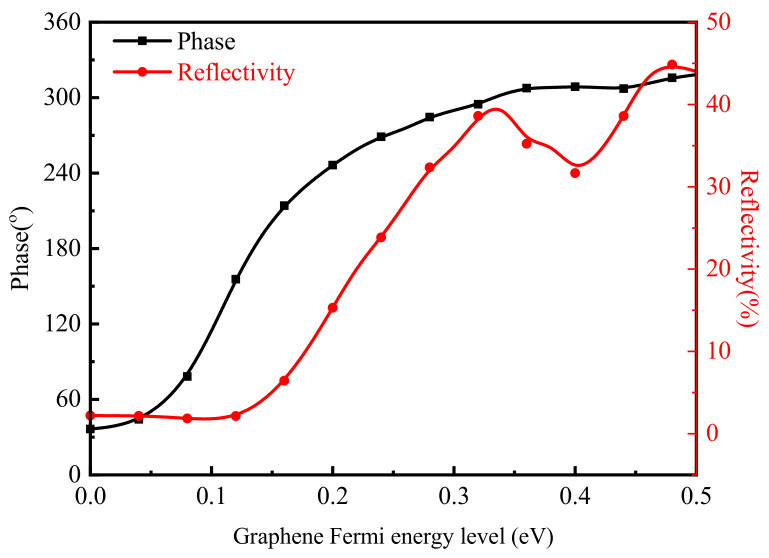
The changing trend of reflection phase and amplitude with various Fermi levels of graphene.

Secondly, the range of phase control and the range of reflectivity variation were simulated. In order to prove the phase control effect of the graphene-metal hybrid metasurface, the relationship between the phase and graphene Fermi energy levels was simulated at a fixed wavelength. Figure 3 shows the phase and reflectivity curves at the wavelength of 6.8 μm which the phase period was changed from –180°~180° to 0°~360° to facilitate subsequent beam deflection design. It can be seen from the changing trend of the black curve in Figure 3 that the phase modulated from 35° to 310° when the graphene Fermi energy level increased from 0 to 0.36 eV. However, the reflectivity of metasurface inevitably changed because of the phase regulation mechanism, which depended on the resonance shift principle. As the red curve in Figure 3 shows, the reflectivity of the metasurface varied by 42%, which would affect the effect of optical deflection control due to the change of the Fermi energy level of the graphene. Therefore, we intend to use the VO_2_ and graphene double control method to control the phase based on constant reflectivity in the future.

**Figure 4:**
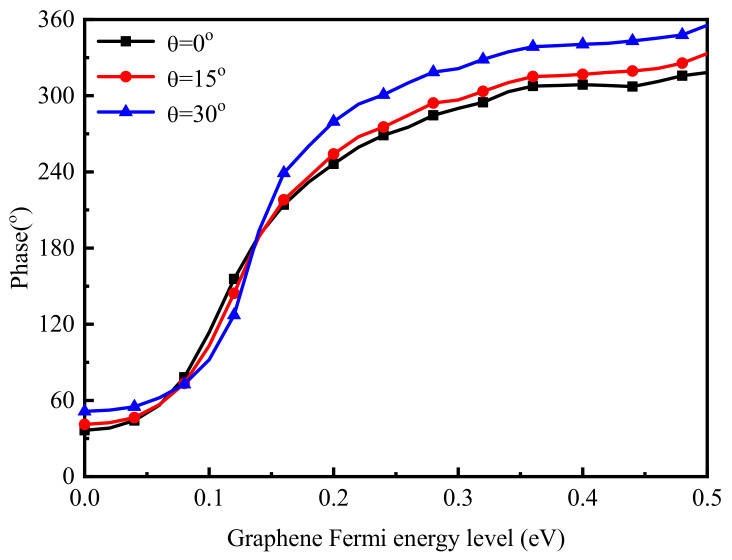
Changing trends of reflection phases at different incident angles with various Fermi levels of graphene.

In addition, the phase modulation characteristics of other wavelengths were also simulated. The simulation results show that the phase control function of the graphene-metal hybrid metasurface could also be realized at other wavelengths. The central wavelength could be changed from 5.8 to 7.7 μm when the size of the long side of the cross increased from 1.9 to 2.4 μm, and the element size remained unchanged. Moreover, the phase control effect could be consistent with the above results by optimizing the thickness of the dielectric layer. Therefore, we can design the corresponding size of the target wavelength according to this characteristic to achieve the characteristics of phase control. Moreover, the phase control curve was almost unchanged, as shown in Figure 4, when the incident angle changed in the range of 30°. As a result, the deflection of the beam at different angles of incidence will be easier to achieve, which is more suitable for reflective OPAs.

**Figure 5:**
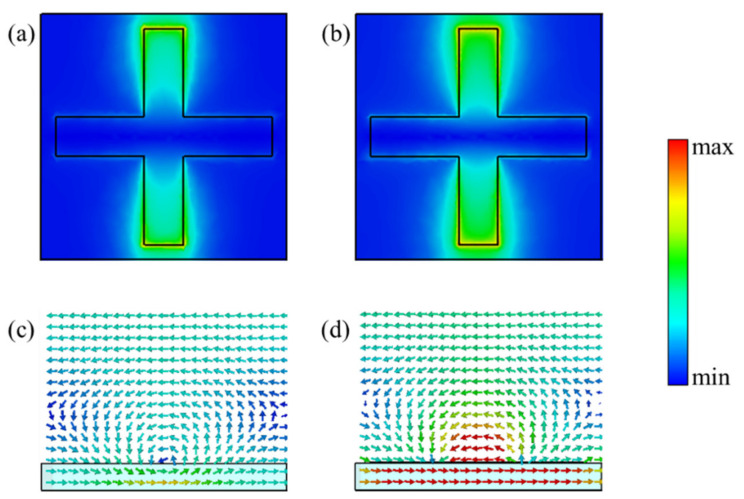
Simulated amplitude distribution of the electric field in reflection mode with (**a**) *μ*_c_ = 0 eV, *λ* = 6.8 μm and (**b**) *μ*_c_ = 0.12 eV, *λ* = 6.8 μm. Simulated cross-section magnetic field distribution in reflection mode with (**c**) *μ*_c_ = 0 eV, *λ* = 6.8 μm and (**d**) *μ*_c_ = 0.12 eV, *λ* = 6.8 μm.

Finally, in order to better understand the resonance phenomenon and the phase control characteristics of the metasurface, the surface electric field distribution and cross-section magnetic field distribution under different Fermi levels of graphene were simulated, respectively. The electric and magnetic field distributions at the graphene Fermi energy levels of 0 and 0.12 eV at the center wavelength of 6.8 μm are shown in Figure 5. It can be seen from Figure 5a that a certain degree of electric field enhancement is generated on the surface of the metal cross, and the electric dipole resonance was formed due to the alternating accumulation of charges at the upper and lower parts of the cross. There was a magnetic resonance, in which the direction was parallel to the opposite direction of the structure surface on the upper and lower surfaces of the metasurface structure, as shown in Figure 5c. The combination of the electric and the magnetic resonances led to the apparent abrupt change of the amplitude and phase of the metasurface at *λ* = 6.8 μm. Moreover, the electric and magnetic field intensities were enhanced while the electric field concentration area and magnetic field direction were the same as before, as shown in Figure 5b,d, when the Fermi level of graphene increased to 0.12 eV. This phenomenon also indicates that the effect of electric and magnetic resonance was enhanced, so that the abrupt phase change was more obvious, which is the reason why the phase was changed, and the center frequency was almost unchanged in the low Fermi level range.

**Figure 6:**
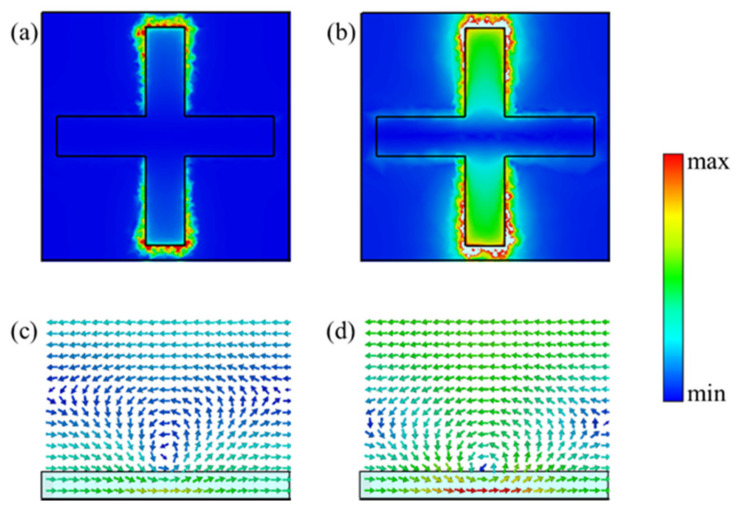
Simulated amplitude distribution of the electric field in reflection mode with (**a**) *μ*_c_ = 0.24 eV, *λ* = 6.8 μm and (**b**) *μ*_c_ = 0.24 eV, *λ* = 6.5 μm. Simulated cross-section magnetic field distribution in reflection mode with (**c**) *μ*_c_ = 0.24 eV, *λ* = 6.8 μm and (**d**) *μ*_c_ = 0.24 eV, *λ* = 6.5 μm.

It can be seen from Figure 6a,c that when the Fermi level of graphene increased to 0.24 eV and the wavelength is still 6.8 μm, the electric field was no longer distributed in the two ends of the cross, but instead in the graphene region near the cross structure. In addition, the magnetic field direction remained unchanged, but the magnetic field intensity was lower than that at 0.12 eV. However, the strong electric and magnetic resonances re-appeared on the metal cross and the nearby graphene surface, as shown in Figure 6b,d, when the central wavelength was 6.5 μm. This also shows that the shift of resonant frequency was the main reason for the phase change when the Fermi level of graphene changed to a higher range.

## 4. Beam Manipulation

**Figure 7:**
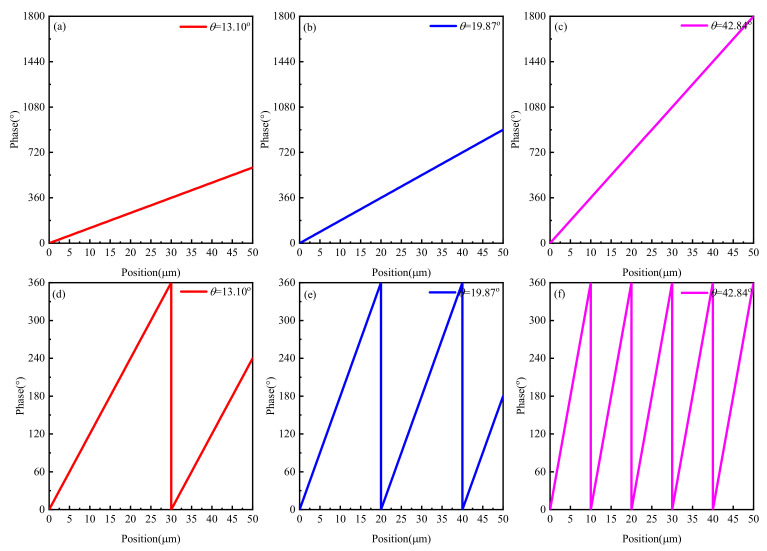
Theoretical results of phases corresponding to deflection angles of (**a**) 13.10°, (**b**) 19.87° and (**c**) 42.84°. Theoretical results of phases corresponding to deflection angles of (**d**) 13.10°, (**e**) 19.87° and (**f**) 42.84° according to a period of 360°.

An optical phased array is a kind of technology to realize beam manipulation by controlling the phase of the unit, so the first thing to do is to calculate the required phase distribution according to the target steering angle. The relationship between phase and steering angle can be expressed as [39]

(4) $$\varphi (x)=k_{0}x\sin\theta =\frac{2\pi }{\lambda_{0}}x\sin\theta ,$$

where *x* is the relative position, *k*_0_ is the wave number, *λ*_0_ is the wavelength of the incident light, *θ* is the target incidence angle, *φ*(*x*) is the phase of the desired position. The results of the phases corresponding to deflection angles of 13.10°, 19.87° and 42.84° are shown in Figure 7a–c, which can be obtained by Equation (4). Then, the curve was re-described as Figure 7d–f, according to a period of 360°.

**Figure 8:**
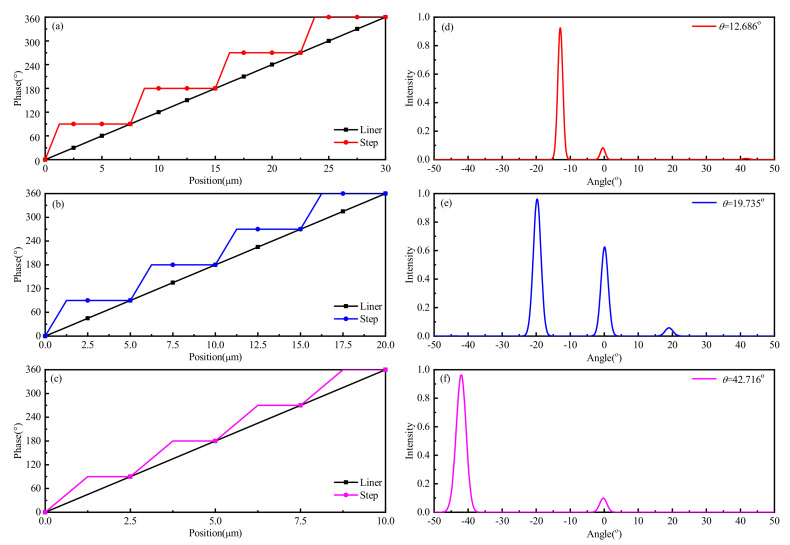
Phase-gradient profile for target beam-steering angles of (**a**) 13.10°, (**b**) 19.87° and (**c**) 42.84°, theoretically. Full-wave simulation results of the beam steering metasurface for (**d**) 12.686°, (**e**) 19.735° and (**f**) 42.716°.

As shown in Figure 8a–c, a 90° phase gradient curve was selected to replace the linear curves of the three different deflection angles. In this process, the *N* column structure units were set as a pixel which was set to the same phase, that is, an equipotential bias voltage was applied. Then, the phases of the adjacent four pixels were set to 90°, 180°, 270°, and 360° respectively, which were regarded as a meta-period and extended to the whole metasurface along this direction. According to the relationship between the steering angle and the phase, *N* was set to 3, 2, and 1, respectively, based on this design principle. It can be seen from the phase curve in Figure 3 that the Fermi energy levels corresponding to 90°, 180°, 270° and 360° were set to 0.09, 0.13, 0.25 and 0.48 eV, respectively.

Figure 8d-f shows the full-wave electromagnetic simulation results of two-dimensional normalized intensity under different reflection angles using FDTD Solutions Lumerical. This device is excited by a linearly *E_x_*-polarized plane wave which impinges at normal incidence with respect to the surface along the z-direction. The dimensions of the calculation space were set to 40 × 2.5 × 5.5 (*x* × *y* × *z*), 30 × 2.5 × 5.5, and 10 × 2.5 × 5.5 μm, respectively. The all-global spatial resolutions were set to 50 nm, where fine resolution (2 nm in each dimension) was used at the graphene-metal hybrid pattern layer. Perfectly matched layer (PML) boundary conditions were used at the edge of the simulation region in the *z*-direction, and periodic boundary conditions are used at the edge of the simulation region in the *x*- and *y*-directions. Finally, the total area of the metasurface was set to 80 × 80 μm in the far-field setting to complete all the settings of the full-wave simulation. It should be pointed out that in the process of OPA simulation, we simulated arrays with area sizes from 20 × 20 to 420 × 420 μm. The simulation results show that the area size is only related to FWHM (full width at half maximum), which will be explained in the last part of this article, and will not affect the beam deflection angle. The reason for taking an area 80 × 80 μm as an example is that the FWHM was significant under this size of an area, so the details of the beam deflection curve and the deflection effect could be observed clearly. It can be seen from the figure that the beam deflection angles were 42.716°, 19.735°, and 12.686° respectively, and the FWHMs which determined the resolution of the OPAs were about 4.79°, 2.62°, and 3.52° under the metasurface phase gradients of *N* = 3, 2, and 1. In addition, there was an obvious side lobe when the deflection angle was 19.735°. It is worth noting that the function of continuous beam deflection can be realized by modulating the value of *N* and that the deflection angle of the beam is positively correlated with the value of *N*. The maximum beam deflection angle of the metasurface was 42.716° when the phase gradient was 90° and *N* = 1. It should be noted that the simulated deflection angles 12.686°, 19.735°, and 42.716° were obtained based on the phase curves calculated from the beam deflection angles 13.10°, 19.87°, and 42.84°, respectively. The difference was due to the metasurface design with its maximum phase range of 270°, and the calculated linear phase curve was stepped because each column unit can only be realized as a phase. Therefore, it is necessary to re-calibrate the relationship between angle and phase in practical application.

**Figure 9:**
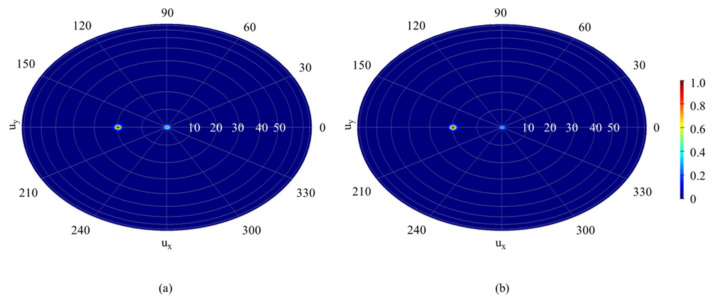
Full-wave simulation far field results of the beam steering metasurface for (**a**) 90° and (**b**) 45° phase gradients.

In order to further explore the effect of phase gradients on the OPAs of the metasurface, metasurfaces of 45° phase gradient and 90° phase gradient with a 19.85° beam steering angle was simulated, respectively. The simulation design method was the same as above for a 90° gradient. However, for the 45° phase gradient, the two-column period was set to one pixel, which was applied to the equipotential voltage. The graphene Fermi energy levels of the adjacent pixels were set to 0.04, 0.09, 0.11, 0.13, 0.18, 0.25, 0.14, and 0.48 eV, in turn. The final result is shown in Figure 9. The quality of the reflected beam was improved due to the decrease of the sidelobe and the original beam deflection angle when the phase gradient decreased. Therefore, a smaller phase gradient can be selected to meet the requirement of high beam quality.

**Figure 10:**
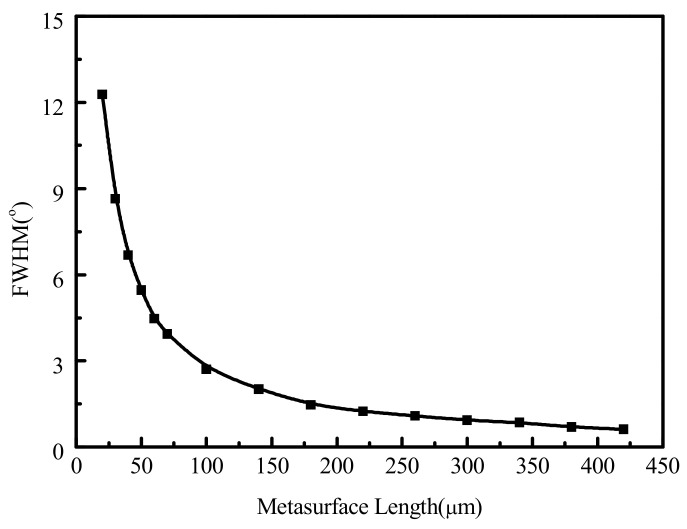
Changing trend of FWHM with various metasurface sizes.

In addition, the FWHM of OPAs is affected by the total size of the metasurface from the simulation results. The FWHM curve with the metasurface size is shown in Figure 10 when the phase gradient was set to 90° and the deflection angle was 42.84°. It can be seen from the figure that the FWHM will decrease sharply to 2.70° at first, and then slowly to 0.62° when the metasurface size increases from 20 × 20 to 420 × 420 μm. This also shows that the angular resolution of OPA can reach about 0.62° at the maximum deflection angle. The resolution will only be slightly improved if we continue to increase the size of the metasurface.

## 5. Conclusions

We showed a promising way to achieve full wavefront manipulation in an MIR regime. With different input voltages designed for a graphene-metal hybrid metasurface, we accomplished modulation of the phase over 275°. The all-solid-state OPA achieved a maximum scan angle of 42.716° and a FWHM of 0.62°. A higher quality beam can be obtained by selecting a smaller phase gradient. In a word, the OPA proposed in this paper can be directly prepared by excellent micro-nano processing technology, which can facilitate the development of metasurface applications, including MIR imaging, optical sensing, and optical communication systems. In the future research work, we will focus on OPA process preparation and batch engineering.

The preparation of graphene will be the most challenging problem to solve in future experiments by analyzing processing in the future. First of all, for the graphene pattern, wet transfer technology will be used to place the graphene film prepared by chemical vapor deposition on the silica dielectric layer, and the graphene grooves will be removed by gas plasma etching. Then, vacuum evaporation coating and photolithography stripping will be used to complete the preparation of the metal cross. Finally, to control the Fermi level of the graphene, the metal substrate will be grounded, and the bias voltage will be applied to the graphene strip, which can be replaced by the cross structure at the tail of each column for simple preparation and stable access.

## Data Availability

Not applicable.
